# A review on herbal interventions and nanotechnological advancements for managing polycystic ovarian syndrome

**DOI:** 10.5935/1518-0557.20250042

**Published:** 2025

**Authors:** Khushi Sharma, Snigdha Bhardwaj, Kandasamy Nagarajan

**Affiliations:** 1 KIET School of Pharmacy, KIET Group of Institutions, Ghaziabad, Delhi-NCR, Uttar Pradesh, INDIA

**Keywords:** polycystic ovary syndrome, physiology, hormonal imbalance, causes, herbals, phytoconstituents

## Abstract

**Objective:**

Polycystic ovarian syndrome (PCOS) is a condition caused by hormonal imbalance that can’t be fully cured, but some symptoms can get better with changes in lifestyle, medications, and fertility treatments. This study aimed to evaluate the efficacy of herbal medicine on ovarian mass, weight, sex hormone disorders, and insulin resistance to treat PCOS. This disorder is mainly characterized by increased levels of androgens, acne, and hirsutism, and can lead to long-term insulin resistance, miscarriage, or even infertility in women. The present study reviews previous studies on the treatment of PCOS using natural drugs and nanotechnology used to treat PCOS.

**Methods:**

A literature search was conducted in the scientific database using the keywords “polycystic ovary syndrome, phytoconstituents, and herbals”. This review includes a collection of reports from Science Direct, Scholar Google, and PubMed were searched up to 2024. The results were assessed, gathered, and reported in this paper. A total of 162 papers were included, with an exponential growth in the number of articles published from 1980 to 2024.

**Results:**

We discuss the significance of herbal remedies in treating PCOS, and the chemical composition, mechanism of action, and therapeutic application of selected herbal drugs against PCOS. These plant compounds can help PCOS patients by improving how their bodies process sugar and making them more sensitive to insulin.

**Conclusions:**

The review suggested that future research should focus on integrating lifestyle modifications and natural therapies to enhance the quality of life for individuals at risk or suffering from PCOS.

## INTRODUCTION

The most prevalent chronic reproductive and metabolic endocrine illness affecting women of childbearing age is polycystic ovarian syndrome (PCOS), which is thought to affect between 4% and 21% of women globally. Hippocrates, who lived from 377 to 460 BC, believed that women with a menstrual cycle of less than three days were strong, had a healthy complexion, and looked male. But they are not concerned about getting pregnant and starting a family. The triangular symptoms of hyperandrogenism, irregular menstruation, and polycystic ovaries define the diverse condition known as polycystic ovarian syndrome. As a result, depending on the illness phenotype, age, and lifestyle of the patient, varied manifestations of this trio may be seen in the patient. Nonetheless, the majority of patients seek medical attention due to menstruation abnormalities and hyperandrogenism’s clinical symptoms (Rababa’h *et al.*, 2022). Serious adverse effects from PCOS can include an elevated risk of endometrial hyperplasia and neoplasia. Moreover, metabolic syndrome, insulin resistance (IR), and low-grade chronic inflammation are extra-reproductive indicators of PCOS (De Leo *et al.*, 2016; Durmus *et al.*, 2017; Rocha *et al.*, 2019). Patients with hyperinsulinemic status have been using metformin extensively to treat ovarian dysfunction, which includes irregular menstrual cycles, reproductive issues, and successive anovulation (Genazzani *et al.*, 2004). However, metformin flatulence and diarrhea are just a couple of the side effects that have been observed when using it within the therapeutic dose range (Kim *et al.*, 2012; Strugaru *et al.*, 2013). Nonetheless, 30% of PCOS-afflicted women will experience regular menstruation. PCOS affects 85%-90% of oligomenorrhea patients whereas 30%-40% of amenorrhea patients will develop PCOS. Up to 70% of women with PCOS have hirsutism, a frequent clinical symptom of hyperandrogenism (Mohd *et al.*, 2019). Studies on the evolutionary significance of PCOS have shown that having numerous follicles is a “fertility storage condition.” Since inflammation is seen as stress, eggs are stored as cysts and released later, when more favorable conditions return, to tide over the inflammation or because the inflammatory female body is thought to be unsuitable for conception. It explains why PCOS individuals frequently have several pregnancies. PCOS exacerbation can be prevented with early diagnosis. A gynecologist can identify PCOS based on a patient’s account of symptoms, including oligomenorrhea in the past, and physical characteristics, such as hirsutism. However, the diagnosis of PCOS is predicated on a set of established standards (Patel, 2018). Uterine fibroids are a similar disorder to PCOS, which affects women’s ovaries. Uterine fibroids, also known as leiomyomas, are benign monoclonal smooth muscle tumors that start in the myometrium. This particular type of round uterine tumor is one of the most prevalent benign tumors. These cancers depend on progesterone and estrogen, but the precise origin of the disease is uncertain. It is challenging to find prevalence before menarche; instead, it typically appears during the reproductive phase. After menopause, prevalence reduces and shrinks significantly (Cramer & Patel, 1990). The bulk of fibroids, which affect 20-40% of women, develop while they are fertile. Eleven to nineteen percent of these occurrences occur in women who are approaching menopause (Munusamy *et al.*, 2017). This leads to a 25 percent prevalence of clinical uterine fibrosis in women. Pelvic pain or pressure, as well as irregular and intense menstrual bleeding, are some of the symptoms of this sickness. Infertility or issues with the local reproductive hierarchy are uncommon (Sweterlitsch *et al.*, 2024).

**Figure 1:**
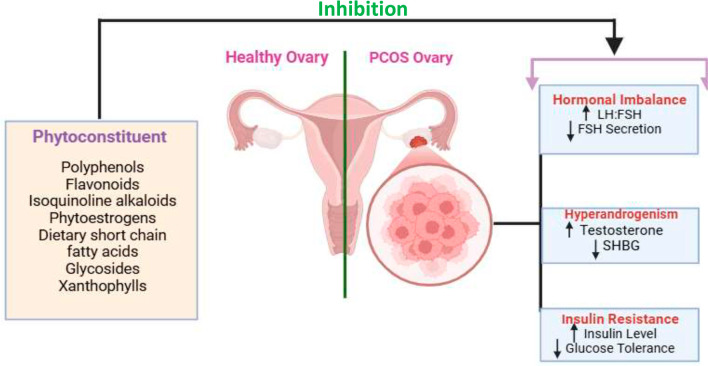
Mechanism of action of Phytoconstituents in managing PCOS.

Phytoconstituents play a significant role in alleviating the symptoms and underlying mechanisms of Polycystic Ovary Syndrome (PCOS) by targeting hormonal imbalances, hyperandrogenism, and insulin resistance through various biochemical pathways. PCOS is characterized by an increased LH:FSH ratio, hypersecretion of androgens, and insulin resistance, which collectively contribute to ovarian dysfunction and the formation of cysts in the ovaries. Phytoconstituents such as polyphenols, flavonoids, isoquinoline alkaloids, phytoestrogens, dietary short-chain fatty acids, glycosides, and xanthophylls work synergistically to modulate these dysregulated pathways and restore ovarian health. Polyphenols, known for their antioxidant and anti-inflammatory properties, help reduce oxidative stress in ovarian tissue, which is a major contributor to follicular dysfunction and cyst formation in PCOS. Flavonoids, by acting as insulin sensitizers, improve insulin signalling and glucose metabolism, reducing insulin resistance and thereby preventing hyperinsulinemia-induced androgen production by the ovaries. Isoquinoline alkaloids exert their effects by modulating neurotransmitter activity and regulating hypothalamic-pituitary-ovarian (HPO) axis function, leading to normalized LH and FSH secretion. Phytoestrogens, plant-derived compounds with estrogen-like activity, bind to estrogen receptors and modulate the endocrine system by lowering androgen levels and increasing SHBG, thereby reducing the effects of hyperandrogenism. Short-chain fatty acids (SCFAs), produced by gut microbiota fermentation of dietary fiber, improve insulin sensitivity and reduce inflammation, contributing to better glucose homeostasis. Glycosides and xanthophylls further contribute to anti-inflammatory and antioxidant effects, reducing systemic inflammation and preventing oxidative damage to ovarian tissue. Together, these phytoconstituents act at multiple levels to restore the hormonal balance, reduce androgen excess, and improve insulin sensitivity, ultimately leading to the normalization of ovarian function and alleviation of PCOS symptoms. By addressing the root causes and modulating multiple pathways simultaneously, phytoconstituents offer a holistic and effective approach to managing PCOS and improving overall reproductive and metabolic health (Figure 1).

## METHOD:STOPHERE

### The general framework of review

In this review, various steps of the framework have been followed which includes

(1) Study Screening
(2) Search strategy
(3) Data extraction

### Study screening

**Figure 2:**
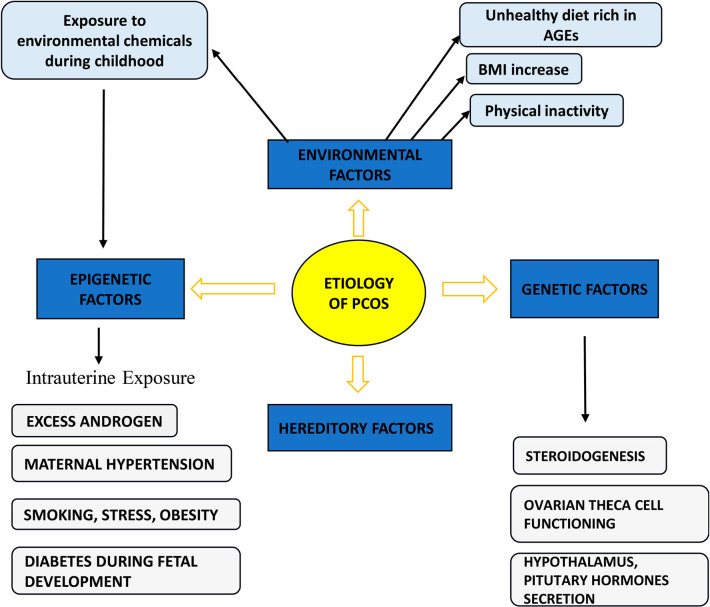
Etiology of PCOS.

Researchers will independently review all relevant studies to identify articles that meet the established inclusion criteria. This process will begin by thoroughly examining the titles and abstracts of the identified studies. To ensure consistency and maintain the scope of the review, only articles published in English, Korean, or Chinese will be considered for inclusion. After the initial screening, the full texts of the selected articles will be carefully analysed to determine their eligibility for final inclusion. In situations where there is a disagreement or discrepancy between the two reviewers regarding the inclusion or exclusion of a particular study, the issue will be resolved through discussion and consensus. If an agreement cannot be reached, the corresponding author will be consulted to make the final decision. To provide a clear and transparent overview of the study selection process, a flow diagram will be used to visually summarize the steps taken, including the number of records identified, screened, and ultimately included or excluded (Figure 2).

### Search strategy

**Figure 3:**
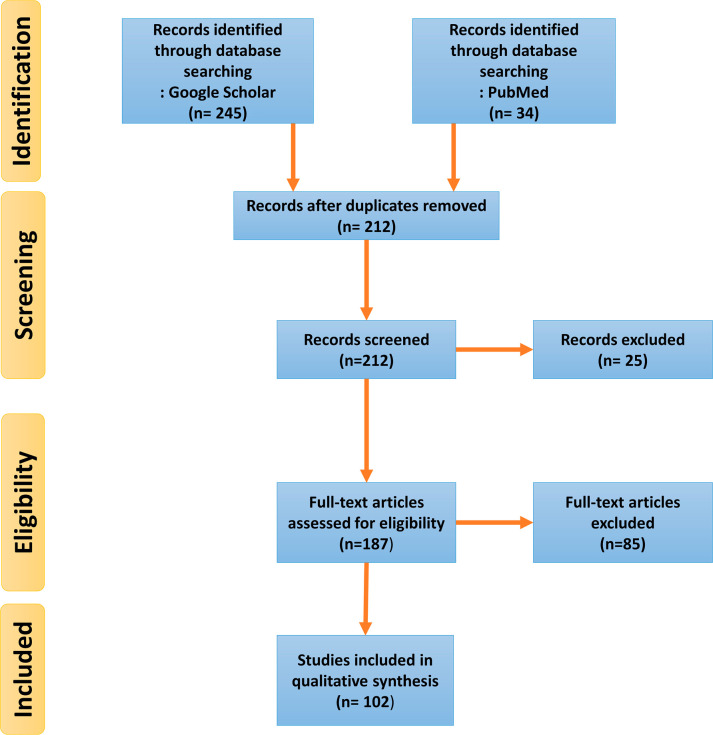
Flow diagram of study selection.

This research presents a comprehensive review of studies conducted between 1980 and 2024 that examine the relationship between polycystic ovary syndrome (PCOS) and various natural interventions, including medicinal herbs, antioxidants, and nutritional approaches. To gather relevant data, an extensive search was performed using international databases such as PubMed, and Google Scholar. The search strategy involved using medical subject headings (MeSH) and combining relevant keywords, including “polycystic ovary syndrome” or “PCOS” along with terms such as “medicinal herbs,” “herbs,” “antioxidants,” “nutrition,” “ovarian cysts,” “hyperandrogenism,” “hirsutism,” “botanical medicine,” and “insulin resistance.” Articles identified through this process, along with references cited in these studies, were carefully reviewed to ensure a thorough and detailed analysis. To maintain the quality and relevance of the review, non-English articles, irrelevant studies, and those deemed inappropriate for the research objectives were excluded. Only studies that quantitatively examined the association between PCOS and the use of herbs or medicinal plants were included in the final analysis, providing a focused exploration of the potential role of natural interventions in managing PCOS (Figure 3).

### Data extraction

Researchers independently scrutinized each eligible article, extracted data and cross-checked the results to ensure the data accuracy. All the paper’s titles, keywords, and abstracts were examined to assess their suitability and applicability for our evaluation. Four separate table in this review describe the Diagnostic criteria of PCOS (table 1), phytoconstituents used against PCOS (tables 2 and 3), and nanocarriers for PCOS (table 4). Data was successfully extracted, and the quality of the data gathered from primary studies was evaluated independently. The authors reached a consensus to settle any disagreements.

**Table 1:** Diagnostic criteria of PCOS.

Diagnostic Criteria	Year of proposal of criteria	Diagnostic features
1. National Institute of Health/ National Institute of Child Health and Human Development	April 1990	1) Hyperandrogenism and/or hyperandrogenism 2) Anovulation or oligo-ovulation 3) The exclusion of a few related conditions, such as congenital adrenal hyperplasia, hyperprolactinemia, and thyroid.
2. Rotterdam criteria European Society of Human Reproduction and Embryology and American Society of Reproductive Medicine	May 2003	Any two of the three traits listed below that describe women: 1) Over-androgenism 2) Oligo-ovulation or anovulation 3) Ovarian polycystic morphology Removal of related illnesses
3. Androgen Excess and PCOS Society	2006	1) Clinical analysis of hirsutism and biochemical analysis of hyperandrogenism involve hyperandrogenemia. 2) Ovarian dysfunction about polycystic ovaries or olig-ovulation/anovulation Removal of overproduction of diseases related to androgen

**Table 2:** Synthetic agents utilize for PCOS treatment.

Category	Name of Drug	MOA	Side Effects	References
**Insulin Sensitizers**	Metformin	Promote the absorption of glucose in intestine, ↓ the liver’s synthesis of glucose, ↓ the absorption of glucose in the intestines, and ↓both fasting and post-meal blood glucose levels.	Diarrhea, Flatulence, bloating, anorexia, stomach discomfort, metallic taste in mouth, and nausea	Mathur *et al*., 2008; Diamanti-Kandarakis *et al*., 2010; Lashen, 2010; Johnson, 2014; Dumitrescu *et al*., 2015; Ryssdal *et al*., 2023
**TZDs**	Pioglitazone	↓ release of fatty acids, ↓ TNF α-induced inhibition of insulin action	Congestive heart failure	Glintborg & Andersen, 2010; Du *et al*., 2012; Madnani et al., 2013; Piątkowska-Chmiel *et al*., 2022)
	Rosiglitazone	↑ transcription of PPAR-γ and ↑ Insulin-induced target cell response	Peripheral Edema	
**DPP-4 inhibitors**	Sitagliptin	↑ incretin levels, ↓ DPP-4 enzyme and ↑ synthesis of insulin by beta cells of pancreas	Angiopathic Edema	Ferjan *et al*., 2018; Modarres *et al*., 2023
	Linagliptin and Alogliptin	Weight increase and hyperinsulinemia	Headache, upper respiratory tract infection	
**GLP-1 agonists**	Exenatide	Weight loss, lipogenesis	Injection site reaction	Bednarz *et al*., 2022
**SGLT-2 Inhibitors**	Empagliflozin	Obesity, androgen excess, Hyperinsulinemia	Infections of urinary system and female genital mycotic	Cassidy-Vu *et al*., 2016; Marinkovic-Radosevic *et al*., 2021
	Dapagliflozin	↓ Renal glucose threshold, ↓ glucose reabsorption from renal tubules	Elevated urination	Elkind-Hirsch *et al*., 2021
	Canagliflozin	↓intestinal reabsorption of glucose, ↑ incretin secretion	Urinary tract disease, moderate diarrhea, nausea, and a female genital mycotic infection	Zhang *et al*., 2022
**Anti-estrogen**	Clomiphene Citrate	Prevents the hypothalamus-pituitary axis from responding to endogenous estrogen in the blood by blocking estrogenic hypothalamic receptors. This, in turn, causes the anterior pituitary to produce FSH in response to changes in GnRH pulsatility.	Impaired vision, nausea and vomiting, breast pain, ovarian enlargement, and abdominal-pelvic discomfort/distention/bloating	Kamath & George, 2011
**Aromatase** **inhibitor**	Letrozole	The enzyme aromatase, which helps turn androgens into estrogens, is competitively inhibited by nonsteroidal compounds.	Headache, hot flashes, asthenia, hypercholesterolemia, edema, bone pain, flushing	Pajai *et al*., 2022
**Antiandrogens**	Flutamide	reduces androgen synthesis through the restoration of ovulation	Hepatocellular Toxicity	De Leo *et al*., 1998; Ryan *et al*., 2018
	Finasteride	impede the activation of DHT by 5 alpha reductases, hence diminishing the impact of androgens.	Sexual dysfunction, depression, erectile dysfunction, loss of libido	Moghetti *et al*., 1994; Traish, 2020
	Spironolactone	Partial blockage of testosterone production, aromatase stimulation, and androgen receptor blockade	Intermenstrual bleeding	Zulian *et al*., 2005; Sabbadin *et al*., 2016)

**Table 3:** Herbal extract used against the polycystic ovarian syndrome (PCOS).

PLANT	EFFECTS	REFERENCE
Curcuma longa	Successfully brought the dropped progesterone levels back to normal and normalized the serum testosterone levels.	Reddy *et al*., 2016
Glycyrrhiza glabra	The LH/FSH ratio dramatically dropped, and FSH levels greatly recovered.	Yang *et al*., 2018a
Ecklonia cava	Returned the hormone levels and regular estrus cycle to normal.	Yang *et al*., 2018b
Aegle marmelos	Serum FSH levels notably decreased and serum LH levels significantly increased.	Dhivya *et al*., 2018
Bougainvillea spectabilis	Serum FSH levels significantly increased while serum LH levels significantly decreased.	Badawi *et al*., 2018
Mateicaria chamomile	The LH/FSH level did not alter significantly.	Heidary *et al*., 2018)
Cinnamomum zeylanicum	Serum total cholesterol levels improved, while serum total antioxidant capacity rose.	Borzoei *et a*l., 2018
Galega officinalis	Considerable drop in FSH and LH levels.	Abtahi-Eivari *et al*., 2018
Moringa oleifera	Significantly higher folliculogenesis and significantly lower insulin levels	Amelia *et al*., 2018
Nigella sativa	Reduction in insulin, FBS, and LH.	Naeimi *et al*., 2018
Vitis	Notable reduction in TC, IL-6, and LDC-C.	Salmabadi *et al*., 2017
Bambusa Bambos	Decrease in triglyceride, levels of glucose, total cholesterol, and very low-density lipoprotein.	Soumya *et al*., 2016
Commiphora weightii	Drop in glucose levels and an increase in hormone profiles.	Kavitha *et al*., 2016
Corylus avellana	Reduction in the levels of FSH and LH	Demirel *et al*., 2016
Palm Pollen	Reduction of LH and elevation of FSH levels.	Jashni *et al*., 2016
Pushpadhanwa rasa	Psychological problems such as headache, irritability, mood swings, sadness, sleep disturbance, lack of confidence, forgetfulness, and libido loss were significantly alleviated.	Dash *et al*., 2016
Saegassum ilicifolium	Increased levels of FSH, LH, estrogen, progesterone, and testosterone in the blood.	Jayaraman *et al*., 2016
Combination of Withania somnifera Dunal and Tribulus terrestris Linn.	Serum FSH levels significantly increased while serum LH levels significantly decreased.	Saiyed *et al*., 2016
Trigonella foenumgraecum	LH and FSH levels have significantly increased.	Swaroop *et al*., 2015
Mentha piperita	Significantly higher amounts of estrogen and significantly lower levels of LH and testosterone. No discernible shift in the FSH level.	Amoura *et al*., 2015
Pergularia daemia	The irregular estrous cycle is reversible.	Bhuvaneshwari *et al*., 2015
Punica granatum	Decrease in the levels of androstenedione hormone, free testosterone, and estrogen.	Jahromi *et al*., 2015
Vitex negundo L	Serum glucose and testosterone levels have significantly dropped.	Shetty Divya & Patil Swati, 2015
Foeniculum vulgare	Serum urea levels have dropped to nearly normal, and Bowman's space and acute tubular necrosis have greatly improved.	Sadrefozalayi & Farokhi, 2014
Alium cepa	Reduction in the amount of cysts and an increase in overall antioxidant capacity levels.	Ghasemzadeh et al., 2013
Aloe barbadensis	Reduction in atretic cysts in the ovaries.	Maharjan et al., 2010

**Table 4:** Nanocarriers for PCOS treatment.

Nanocarrier	Key Ingredients	Therapeutic Agent	Research Object	Mechanism	References
Chitosan Nanoparticles	Chitosan	Curcumin	Rat	Lower the levels of prolactin, serum luteinizing hormone, Insulin and testosterone	Raja *et al*., 2021
Ginger Nanoparticles	Lipid	Ginger	Mice	enhanced the forkhead’s expression Utilize the transcription factor (Foxa2) to lessen insulin resistance caused by intestinal epithelial cell (IEC) exosomes.	Kumar *et al*., 2022
Silver Nanoparticles	Silver	Cinnamomum cassia	Rat	Antioxidant	Kouame *et al*., 2019)
Silver nanoparticles	Silver	Cinnamomum zeylanicum	Rat	Reduce the levels of inflammatory markers such as TNF-α, IL-6, and IL-18	Alwan & Al-Saeed, 2023
Iron nanoparticles	Iron oxide	Curcumin	Mice	prevention of ovarian damage cell death and Cell death was brought on by dehydroepiandrosterone	Fatemi Abhari *et al*., 2020
Selenium nanoparticle	Chitosan	Selenium dioxide	Rat	Cut down on androgen production to stop the vicious cycle. resulting from decreased expression of androgen receptors due to high androgen release	Abdallah *et al*., 2023
Selenium nanoparticle	Chitosan	Selenium dioxide	Rat	Increased expression of the PI3K and Akt genes decreases Sex hormone levels, lipid profiles, insulin sensitivity, inflammation, oxidative stress, and indicators of mitochondrial function	Rabah *et al*., 2023
Liposomes	Glycerol phospholipid	Methoxy derivatives of resveratrol (DMU-212)	Ovarian granulosa cells	Elevate progesterone and estradiol secretion	Józkowiak *et al*., 2023
Carbon nanotubes	Silkworm powder	Nitrogen-doped carbon nanorods (N-CNR)	Mice	Lower fasting blood sugar levels and enhance serum biomarkers levels linked to inflammation and oxidative damage	Park *et al*., 2022
Quantum dots	Polyethylene glycol	Metformin	Hepg2 cells	Reverse insulin resistance and restore glucose absorption.	Sarkar *et al*., 2024
Micelle		Curcumin	Rat	Decreased oxidative stress and inflammation responses	Nazarian *et al*., 2025

## PATHOPHYSIOLOGY

PCOS has a high occurrence rate, however its etiology is yet unknown. Owing to the variation in how clinical and biochemical aspects are represented, there has been discussion over whether PCOS genuinely represents a single condition or multiple ones. PCOS symptoms often appear during puberty, however the underlying cause may have been preprogrammed as early as fetal development. Insulin resistance, which affects 85% of PCOS-afflicted women, is one of the condition’s most prevalent characteristics. Elevated androgen levels, another typical symptom of PCOS, affect 60-80% of PCOS women and can cause hirsutism, acne, and, to some extent, alopecia. Gas chromatography has shown elevated amounts of circulating estrogens, androgens, sex steroid precursors, and glucuronidated androgen metabolites in PCOS (Sabuncu *et al.*, 2001; Johansson & Stener-Victorin, 2013; Aversa *et al.*, 2020).

### Hyperandrogenism

An excess of androgen hormones is one of the main PCOS symptoms and the development of metabolic syndrome. These androgens are mostly produced by the ovaries and adrenal glands. Although roughly 20-30% of women with PCOS may have adrenal hyperandrogenism, this condition’s metabolic problems are not greatly impacted by it; instead, the primary pro-inflammatory hormone, aldosterone, frequently rises when adrenal hyperandrogenism occurs (Schindler *et al.*, 2008; Na *et al.*, 2022; Stańczak *et al.*, 2024). However, one major element that contributes to the development of PCOS is the ovaries’ overproduction of androgens. Understanding androgen production, particularly in the ovaries of women with PCOS, and analysing its effects at the cellular level will be the main topics of the sections that follow (Ye *et al.*, 2021).

### Insulin resistance

Insulin resistance is a pathological condition of the body that is defined by reduced insulin biological effects, even at high concentrations, which causes problems with glucose uptake and transportation. One of the pathological elements connected to metabolic abnormalities is IR in female PCOS patients. There is a prominent relationship between testosterone, insulin resistance, and hyperinsulinemia, and Burghen *et al.* (1980) made the initial discovery of androstenedione levels in PCOS patients. The insulin phenotype, hyperandrogenism, resistance, and hyperinsulinemia are all brought on by mutations in the insulin receptor gene (Moller & Flier, 1988; Siddiqui *et al.*, 2022). Although insulin resistance and hyperinsulinemia are frequently observed in PCOS patients, uncommon mutations in the insulin receptor gene have been discovered in female PCOS patients (Witchel *et al.*, 2019). For whatever reason, excessive body mass and insulin resistance indices are unrelated to one another because it has been shown that thin women with PCOS diagnoses also have insulin intolerance. Recent assessments show that whereas 20% of women with insulin-resistant PCOS are slender, over 80% of them are overweight (Toosy *et al.*, 2018; Aversa *et al.*, 2020).

### Oxidative Stress

The pathophysiology of PCOS is associated with oxidative stress, which occurs when the body generates reactive oxygen species (ROS) more quickly than antioxidants can eliminate them. Leukocytes in peripheral blood have been shown in numerous investigations to produce ROS in response to hyperglycemia (González *et al.*, 2006). Hyperandrogenism, insulin resistance, and an increased risk of heart-related disorders are caused by increased oxidative stress and the release of pro-inflammatory cytokines (Victor *et al.*, 2009; Aversa *et al.*, 2020). According to several studies, PCOS women’s serum has elevated levels of oxidative stress indicators, and this state is also present in the follicular fluid of oocytes (Liu *et al.*, 2021). Increased oxidative stress markers in oocyte follicular fluid can also lead to abnormal follicle growth and development, as well as low-quality oocytes or embryos, which can make it impossible to conceive. DNA oxidative damage is also associated with carcinogenesis. Superoxide, hydroxyl, and hydrogen peroxide are examples of reactive oxygen species that have the potential to harm DNA (Ziech *et al.*, 2011).

## NECESSITY TO STUDY PCOS

**Figure 4:**
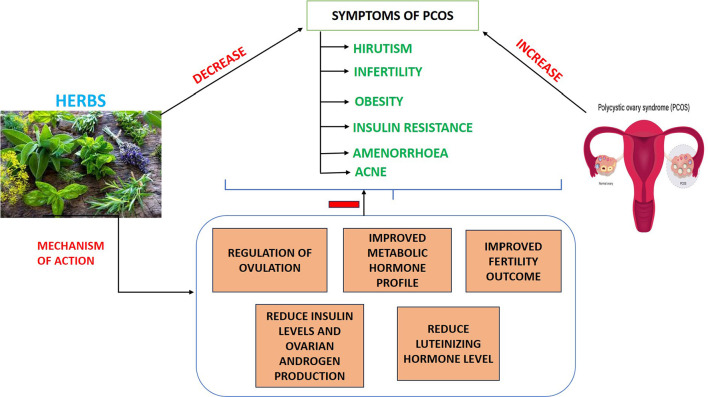
PCOS Symptoms.

Six out of ten women with PCOS are teenagers, according to research by the PCOS Society. 20 to 25% of women in their reproductive age have PCOS, according to a study by the AIIMS Department of Endocrinology and Metabolism. Although 35-50% of women with PCOS have fatty livers, 60% of them are obese. About 60% to 70% of adults have high testosterone levels, 40% to 60% are glucose intolerant, and 70% are insulin resistant. Although the exact cause of PCOS is unknown, some women with the condition have higher-than-normal insulin levels. Excessive insulin production can lead to the ovaries producing higher levels of androgens, including testosterone. Because insulin resistance might make it more difficult to shed weight, PCOS-afflicted women frequently deal with the obesity problem. It’s risky because, among other long-term health issues, undetected or poorly treated conditions might result in infertility. A variety of symptoms, including weight gain, exhaustion, unwelcome hair growth, hair thinning, infertility, acne, pelvic discomfort, migraines, sleep issues, and mood swings, are displayed by girls and women with PCOS (Figure 4). On the other hand, it results in diabetes mellitus, hypertension, and aberrant blood lipid levels in elderly people. Studies comparing allopathy, ayurveda, and homeopathy show that while homeopathy and ayurveda promise no side effects and are the best treatments, allopathy does not cure PCOS; instead, it helps manage and control its symptoms and demands more time and money. Thus, research is increasingly necessary (Khanage *et al.*, 2019).

## SYNTHETIC TREATMENT

After lifestyle therapy for PCOS, pharmacological medicine is frequently utilized as subsequent therapeutic approach. The prescription choice is influenced by the patient’s primary concerns, conceiving objectives, and any coexisting medical conditions, such as type 2 diabetes. insulin sensitizers, oral contraceptives, and anti-androgen medications are a few examples of potential therapeutic substitutes. For the treatment of acne, combination oral contraceptives (COCs) are superior to progestin-only formulations and hirsutism, as well as their potential to cure irregular menstruation (Chakravorty, 2023).

## HERBS USED IN PCOS

The term “herb” is frequently used in commerce to describe a plant, plant component, or extract used for flavour, aroma, or medical purposes. Herbal remedies are naturally occurring substances that have had minimal to no industrial processing and have been used traditionally to treat a wide range of illnesses. Traditional herbal remedies are receiving a lot of attention in discussions about global health. Traditional medicine has a well-established function in therapeutic, rehabilitative, and preventive care (Knochenhauer *et al.*, 1998). Given that they typically cause less adverse effects than medication and are generally relatively soft on the body, herbal remedies can be a very efficient PCOS treatment choice. The use of herbal therapy has peaked. It is vying for recognition as a science, a distinct field unto itself. It is now required to demonstrate that herbal rehabilitation is on par with other medical specialties in terms of the breadth of its scientific research and its application in real-world scenarios. Herbal medicine has several advantages over conventional therapy, including safety and fewer adverse effects. Additionally, the presence of several active substances in medicinal plants results in a potentiating effect (Jungbauer & Medjakovic, 2014). The capacity of herbs to be used for longer periods with fewer adverse effects is crucial because PCOS demands a protracted administration. They might prove useful in treating the root causes of PCOS, reducing symptoms, and promoting healing by boosting your immune system. We are able to combine a PCOS-friendly diet with natural treatments. Proper workout regimen to increase the efficiency of the selected herbal remedy (Sasikala & Shamila, 1992).

### The Constraints of Herbs Usage

#### Reproducibility of Biological Activity of Herbal Extracts

One of the main barriers to using plants for pharmacological discovery is the lack of repeatability of activity in over 40% of plant extracts (Bandaranayake, 2006). The main reason for this problem is that when plants are harvested and extracted again, the biological activity seen in the first screenings frequently does not occur again. These disparities are mostly caused by changes in the biochemical profiles of plants, which can vary depending on a number of variables, including the time of harvest, the location, the type of plant, and variations in extraction techniques and biological activity tests. Furthermore, complex interactions between several components-whether additive or synergistic-often result in the therapeutic benefits of plant extracts. Therefore, it is important to assess the qualitative and quantitative variations in the bioactive phytochemicals present in plant materials using a comprehensive methodology. The amount of bioactive secondary metabolites can be significantly influenced by a variety of agroclimatic factors, such as location, climate, microenvironment, and physical or chemical stimuli (also known as elicitors). The constancy and dependability of plant extracts in drug discovery may be significantly improved by elucidation-induced increases in these metabolites, which could otherwise go unreported in screening. It may be feasible to create herbal medicines in a way that is both economical and quality-controlled, enhancing their effectiveness and reproducibility in pharmaceutical applications, by concentrating on standardization, optimization, and exact control of growing conditions (Cordell, 2000).

#### Toxicity and Adverse effects

Although herbs are “natural,” many people believe they are safer than prescription medications. But the truth is more nuanced. Like other medications, healing herbs are not always risk-free or dangerous. In small doses, they may have little effect, while in the correct amounts, they can provide therapeutic benefits. On the other hand, when used in excessive amounts or over long periods, they can cause harm. The toxicity of herbal medicines can arise from several factors. First is errors in plant identification, which can lead to the use of the wrong species. Second is the accidental consumption of plants with cardiotonic properties, which can affect heart function. Third is inappropriate combinations of herbs, especially when toxic plants are used together or with other medications. Fourth is the presence of compounds that interfere with conventional drugs, such as those found in plants containing coumarin derivatives, high levels of tyramine, estrogenic substances, or those that cause allergic reactions, skin irritation, or photosensitivity. Recent research has shown that many herbs traditionally considered safe can actually be toxic, with some being mutagenic or even carcinogenic (Schimmer *et al.*, 1988; 1994; Sá Ferreira & Ferrão Vargas, 1999). Therefore, determining the safety of herbal drugs requires careful consideration of factors such as purity, the presence of harmful substances, bioavailability, and any known adverse effects. These elements are essential for setting safe use guidelines for herbal remedies.

#### Adulteration and Contamination

In nations with lax laws governing the integrity of herbal remedies, adulteration and contamination are common. Because these adulterations are frequently unanticipated and can go unnoticed unless linked to an outbreak or epidemic, this is especially problematic. Veno-occlusive illness is one risk that can arise from eating plants that contain pyrrolizidine alkaloids. This illness may be severe, potentially lethal, or even life-threatening (Drew & Myers, 1997). Herbal medications that are tainted or contaminated can cause major health issues, especially in young patients (Ernst & Thompson Coon, 2001; Saper *et al.*, 2004). Thirteen incidences of heavy metal toxicity in children who utilized herbal medicines were reported between 1975 and 2002, according to a recent assessment. These cases started in Singapore, Hong Kong, the United States, the United Kingdom, and the United Arab Emirates.

When environmental pollution and the inorganic chemicals used in some Ayurvedic remedies are combined, the levels of heavy metals can rise above acceptable limits, particularly in wealthy nations. In Asian medicines, adulteration often happens due to plants being misidentified, which can lead to poisoning from harmful plants like digitalis, belladonna, or skullcap (Elvin-Lewis, 2001). The California Department of Health discovered in 1998 that heavy metals or unreported pharmacological substances were present in 32% of Asian patent medications marketed in the United States (Ko, 1998; Marcus & Grollman, 2002). Prescription medications such as glyburide, sildenafil, colchicine, adrenal steroids, and alprazolam have also been found by the FDA and other agencies in goods that were meant to only include natural ingredients (Ernst, 2002).

#### Herb-Drug Interaction

Herbal medicines can affect the way the body processes other drugs, altering their pharmacokinetic properties (Fugh-Berman, 2000). For example, St. John’s wort accelerates the breakdown of a variety of medications by stimulating intestinal P-glycoproteins and the cytochrome P450 enzyme CYP 3A4. This can reduce the effectiveness of medications like cyclosporine, antiretroviral drugs, digoxin, and warfarin (Moore *et al*., 2000). There are many examples of interactions between drugs and herbal medicines. These interactions can impact the patient’s general metabolism, improve or impair drug absorption or metabolism, or result in unfavourable side effects like hypersensitivity or allergic responses (Cupp, 1999). When someone is receiving treatment for problems including diabetes, depression, pain, asthma, heart disease, high blood pressure, weight loss, anticoagulant therapy, or depression, it’s critical to carefully evaluate the impact of foods and herbal medicines. The scientific evidence on how different herbal medicines interact with drugs, as well as their impact on drug metabolism and bioavailability, should be thoroughly assessed. This helps evaluate both the potential risks of toxicity and the pharmacological foundation for their effectiveness (Bhattaram *et al*., 2002).

#### Regulatory Challenges of Asian Herbal Medicines

In general, most herbal remedies have a far lower rate of severe adverse responses than pharmaceutical medications. To curb unethical activities, however, stricter oversight of traditional medicine makers and practitioners-including those with Indian roots-is still required. Many herbal products are difficult, if not impossible, to verify for purity and safety after processing. This issue is even more challenging for traditional medicines imported from Asian countries, where the use of potentially harmful herbs or heavy metals could not be considered harmful in their home country. Countries like the US and Canada have rejected some Chinese and Ayurvedic medicines from India because they contain excessive amounts of heavy metals and other harmful ingredients. In response to these worries, “Ayush,” India’s traditional medicine regulating authority, has put in place stringent rules governing the export of all herbal remedies, including those derived from Siddha, Ayurvedic, and Unani systems (Bandaranayake, 2006).

## NANOTECHNOLOGY USE IN PCOS

Liposomes and nanoparticles are examples of nano-sized carriers that present a possible means of enhancing the efficacy and administration of PCOS drugs. Hydrophobic medications can be encapsulated by these carriers, which facilitates the body’s absorption. Additionally, they prevent the medications from degrading or metabolizing too rapidly, enabling more of the active ingredient to reach its intended location. Nanoscale carriers can be designed to assemble specifically in the affected tissues or cells by PCOS, which helps minimize side effects on healthy tissues. Furthermore, controlled-release formulations can minimize the need for frequent dosage and enhance the drug’s long-term effects by extending the drug’s half-life in the bloodstream. This implies that the intended therapeutic benefits can be obtained with fewer dosages of drugs. Drug costs can be decreased and adverse effects that are frequently associated with large dosages can be reduced by lowering the dosage of medications utilizing nano-sized carriers. These carriers can also deliver multiple drugs or treatments at once, which is particularly helpful for treating the complex symptoms and causes of PCOS. By delivering drugs straight to the affected areas and preventing their interaction with unrelated tissues, nanoscale carriers lower the likelihood of toxicity and adverse effects. Furthermore, by lowering the number of dosages required, these carriers can streamline treatment plans and make it simpler for patients to adhere to their recommended schedule. Improved patient compliance and treatment adherence may result from this ease. According to a person’s PCOS symptoms, hormone levels, and response to treatment, nano-sized carriers can even be tailored to suit their unique requirements. Further advancements in nanotechnology research may result in even more precise carriers that react to bodily events, enhancing the delivery and targeting of medications. However, before these techniques can be widely utilized to treat PCOS or other medical disorders, more preclinical and clinical research is required to prove their safety and efficacy (Singh *et al.*, 2024). Assisted Reproductive Technologies (ART) could be revolutionized by nanotechnology, which could also aid with animal health and fertility issues, such as those faced by PCOS-affected women (Shandilya *et al.*, 2020; Abruzzese *et al.*, 2022; Na *et al.*, 2022). Targeted drug delivery solutions for PCOS treatment are being developed using nanotechnology. Creating nanoparticles that can directly carry drugs or hormones to the ovaries, it reduces overall side effects and enhances the effectiveness of the treatment (Javid-Naderi *et al.*, 2023). Nanoscale diagnostic technologies could improve early PCOS identification and surveillance. Hormonal balance can be maintained by creating nanoparticles that release hormones like insulin, LH, or FSH under controlled conditions. Nanotechnology may eventually enable targeted gene therapy to address the genetic reasons of PCOS, though this is currently being researched. By encouraging tissue regeneration, nanotechnology may help women with PCOS-related ovarian damage by controlling the release of growth factors and other bioactive substances. Nanotechnology may make it possible to create insulin-releasing implants for women with PCOS-related insulin resistance, which would enhance their metabolic health and reproductive results. Nanoparticles may also be used in gene editing or gene transfer to treat the genetic causes of PCOS and maybe stop the condition from occurring in subsequent generations. Although ART treatments can occasionally result in multiple pregnancies, nanotechnology-based techniques may help increase the success rate of single embryo transfers and reduce the risk of multiple pregnancies. By examining each patient’s own genetic and hormonal profile, nanotechnology may also enable customized therapy regimens, increasing the likelihood of positive results for PCOS-afflicted women. Nanoparticles may also enhance cryopreservation techniques for eggs and embryos, boosting post-thaw survival rates and preserving the potential for reproduction. Drug delivery methods based on nanoparticles have generated a lot of interest because of their potential to improve treatment precision and efficacy (Chandrakala *et al.*, 2022). Nanoparticles can reduce adverse effects and the necessary dosage by directly delivering drugs to the ovaries or other affected tissues (Carissimi *et al.*, 2022). The collection of nanoparticles at the desired location may be enhanced by active targeting using ligands that bind to receptors that are highly expressed in PCOS tissues (Khetan *et al.*, 2022). To help treat hormonal imbalances, nanoparticles might be designed to carry hormones, such as anti-androgens or insulin-sensitizing chemicals, straight to the ovaries or other impacted organs (Lakshmi, 2013). One typical feature of PCOS is insulin resistance. Insulin-sensitizing medications, including metformin (Glucophage), could be delivered to the ovaries directly via nanoparticles, increasing their efficacy and lowering systemic side effects (Rabah *et al.*, 2023). One of the earliest oral antihyperglycemic medications, metformin (Glucophage), is undergoing a reformed formulation to enhance its pharmacodynamic and pharmacokinetic characteristics. Metformin formulations based on nanotechnology have greatly enhanced the care of diabetic patients. Furthermore, metformin-loaded polydopamine nanoparticles have demonstrated significant promise as a possible monotherapy for the prevention and treatment of Parkinson’s disease because of their substantial neuroprotective properties. Notably, nanomedicine is emerging as a powerful approach for future use in clinical settings across various diseases (Chen *et al.*, 2020). Another element that contributes to PCOS is inflammation, and anti-inflammatory nanoparticles may be targeted to the reproductive system’s inflammatory regions (Dimik *et al.*, 2023). Nanoscale systems also enable combination therapies, allowing multiple medications to be delivered in a single formulation. This strategy might be very helpful in tackling the complexity of PCOS (Thapa *et al.*, 2022). By eliminating the need for frequent dosages and enabling regulated, slow medication release, nanoparticles may enhance patient adherence and the overall effectiveness of treatment. To make sure these nanoparticle systems don’t damage other tissues or organs, scientists must carefully assess their long-term biodistribution and safety. Considering the eventual goal of commercializing these treatments, it is likely that developments in nanoparticle-based drug delivery systems for PCOS therapy have advanced to the preclinical and clinical stages. Nanotechnology offers the potential to minimize harm to healthy cells while precisely targeting damaged regions and receptors (Sim & Wong, 2021).

## CHALLENGES FACING WITH NANOTECHNOLOGY

The current era, sometimes known as the “no or low controversy era,” has seen an all-time low in fear or resistance to new technologies. Because of this, new technologies like nanotechnology are typically welcomed with great enthusiasm (Satterfield *et al*., 2009). However, successful sales are not assured by merely presenting a newer product or gaining technological knowledge. The industry must completely adopt these advancements if it is to prosper. It is essential for the industry to have confidence in the technology’s performance. Only then will it be able to direct its efforts toward gaining public support. In this context, it seems several challenges are hindering the successful commercialization of nanotechnology-based products. The two primary requirements for such products are ensuring they are both effective and safe for use. While many nanotechnology-based products are developed, they sometimes fail to meet one or both of these criteria, which prevents them from reaching the market successfully (Mazzola, 2003; Morigi *et al*., 2012).

### Safety

The USFDA’s advisory states that the main regulatory issue is not therapeutic failure but rather test product safety, particularly when the test product’s plasma levels are significantly greater than those of the reference product, as is frequently the case with systems based on nanotechnology. These products frequently face challenges related to bioaccumulation, excessive bioactivity due to their nanosize, metabolism, elimination from the body, and biocompatibility during use. Furthermore, some studies have raised concerns about their overall suitability for human use (Vega-Villa *et al.*, 2008; Bosetti & Vereeck, 2011). Recent studies conducted by Chinese researchers have raised concerns regarding the absorption of nano-silver during animal tests, suggesting it may interfere with DNA replication and alter molecular networks, potentially causing genetic modifications. Nano-silver is commonly incorporated into materials used in food packaging, among other applications, as it helps extend the shelf life of food by eliminating harmful microorganisms (Suppan, 2011). These findings have contributed to a growing negative perception of products containing nano-silver. Potential cellular interactions of nanoparticles that could cause cytotoxicity and other biological reactions, as seen from a mechanistic standpoint, include: (1) relationships with the plasma membrane, resulting in instability that impacts signal transduction, ion transport, and may cause cell death; (2) interactions with mitochondria, altering metabolism or disrupting antioxidant defences (3) attaching to DNA, harming it, stopping the cell cycle, and preventing the creation of proteins; (4) interacts with the cytoskeleton, which can cause mechanical instability, interfere with vesicular trafficking, and cause cell death; and (5) interacts with other substances, including lipids and proteins. As demonstrated by cyanoacrylate nanoparticles, nanoparticles can also have cytotoxic effects by adhering to the cell membrane, degrading there, and then releasing poisonous breakdown products (Lherm *et al.*, 1992).

### Reproducibility

A pharmaceutical product is considered to have reached the market when each batch produced consistently exhibits the same physical characteristics and therapeutically significant properties. For products utilizing nanotechnology, it is essential that they also adhere to these standards, ensuring minimal variations between batches. This is particularly important to guarantee that the product delivers the same performance and appearance in every batch, as consistent quality is crucial for customer satisfaction. The verified manufacturing process must incorporate this reproducibility into the nano drug delivery system (Mojahedian *et al.*, 2013). Nanoparticles can be highly sensitive to even small changes in operational conditions, which can often lead to an increase in size, causing the particles to fall outside the desired or specified nano range (Gaumet *et al.*, 2008). Changes in crystallinity, drug entrapment and release profiles, and surface charge are some of the additional variables that may be impacted throughout production, even though size changes are among the most obvious. The formulation and its intended performance may be greatly impacted if these variances are not handled. Moreover, biologically active materials that are especially prone to degradation, such as proteins and nucleic acids, are present in many nano formulations. As a result, these products require careful handling during manufacturing to preserve their integrity. This article highlights the difficulty of managing “batch-to-batch” variability in industrial production of products based on nanotechnology. The ideal approach is to use a robust preparation method that can accommodate a broad range of operational conditions without causing significant changes to the formulation, even if deviations occur due to human error or equipment malfunction within an acceptable range. Furthermore, thorough in-process testing is necessary to guarantee the formulations’ overall integrity, bioactivity, and uniformity between batches (Langer *et al.*, 2008).

### Characterization and Quality Control check

Building on the last point, it’s critical to specify the characterization techniques and industry perceptions of products based on nanotechnology. Strict quality control procedures must be incorporated into each step of the production process for these goods, just like for other pharmaceuticals. These analytical checkpoints play a key role in ensuring the quality and consistency of each batch, which is essential for the successful launch of any pharmaceutical product (Desai, 2012). Standard analytical tools like spectrophotometers, pH meters, hardness testers, and dissolution apparatus are commonly used in industries. To describe nanoparticles, however, more intricate and advanced techniques are required, such as atomic force microscopy, transmission electron microscopy (TEM), and scanning electron microscopy. Capillary electrophoresis, analytical ultracentrifugation, and the use of both static and dynamic light scattering to determine particle size and size distribution are more methods. In addition to surface charge or zeta potential measurements, surface chemistry analysis is carried out using techniques like X-ray diffraction, differential scanning calorimetry, X-ray photoelectron spectroscopy, and Fourier transform infrared spectroscopy (Mäder, 2020). These analytical methods and equipment are expensive, and they need skilled workers to conduct the investigations and interpret the findings. Therefore, an industry needs both specialized analytical equipment and a trained staff of professionals to run and maintain these tools in order to enable the manufacture of products based on nanotechnology (Desai, 2012). This would considerably increase manufacturing costs, likely deterring companies from investing in the development of such products. Even if a company chose to outsource these evaluations, costs would still be high, as every batch would need to undergo multiple tests, and transportation to the testing facility would be required. One potential solution to this challenge is to establish analytical hubs near emerging industrial areas, making advanced characterization and analytical methods more accessible to these sectors. Governments supporting the creation of such a collaborative industrial ecosystem would undoubtedly help foster the growth of the nano-based pharmaceutical industry.

### Translation into Suitable Dosage Form

The most popular and well-known solid oral dose forms worldwide are tablets and capsules. Most industries already have established infrastructure for manufacturing these forms. However, many nanotechnology-based products are typically produced as water-based dispersions. Converting these liquid systems into solid oral forms presents a significant challenge. To create a solid or semisolid powder, large volumes of nanoparticles would need to be lyophilized, or freeze-dried. The resultant powder after lyophilization can be compressed into tablets or put into capsules, with additional excipients added if necessary. However, lyophilization is an expensive process, especially when performed on a large industrial scale. One of the challenges is that nanoparticles may aggregate and increase in size during this process, and this change may not be fully reversible when the product is reconstituted (Saez *et al.*, 2000). During lyophilization, cryoprotectants are frequently added; however, this can change the formulation’s drug release characteristics (Fonte *et al.*, 2012). Direct lyophilization of nanoparticulate systems may not be feasible, and additional steps like centrifugation or diafiltration might be required. These extra processes increase the complexity of the manufacturing procedure and make large-scale production less viable. Moreover, it will be necessary to maintain the stability, biological activity, and integrity of the nanoparticles in the final product. Direct lyophilization of the nanoparticulate system may not be feasible, and instead, processes like diafiltration or centrifugation may be needed. These additional steps complicate the procedure and make large-scale production less practical. Moreover, ensuring the stability, biological activity, and integrity of the nanoparticles in the final product is crucial. As a result, converting aqueous nanoparticle dispersions into solid oral formulations demands substantial technical investment.

### Lack of multidisciplinary platform

Nanotechnology products are part of an interdisciplinary field that combines nanoscience, nanoengineering, and nanotechnology with life sciences (Mazzola, 2003). As a result, a transdisciplinary approach is crucial for the creation, assessment, and successful commercialization of these products in addition to the original concept (Heimeriks, 2012). No one specialist have all the abilities needed to independently launch a product using nanotechnology. Furthermore, scientists frequently lack the business savvy needed to turn technology into a product that can be sold. However, investors frequently lack the technical know-how and patience required for the creation and assessment of nanotech-based goods, even though they want to be a part of the next big breakthrough (Mazzola, 2003; Flynn & Wei, 2005). Moreover, when these products are approached from a single-disciplinary perspective, they become quite complex, especially when an individual tries to address issues and specific needs outside their area of expertise. The challenges faced during product development would likely be better addressed if a diverse team of formulation scientists, economists, statisticians, engineers, and chemists, along with other academics and researchers, collaborated to engage in thorough discussions and develop theories related to these products (Mowery, 2011). In light of this, the National Institutes of Health (NIH) has created a roadmap for the “future of nanomedicine,” offering financial support to organizations that integrate several fields to successfully address the different issues related to products based on nanotechnology (Kantor, 2008).

### Public

The public perception of nanotechnology-based products is critical for their successful marketing. Unfortunately, despite their significant potential health benefits, these products remain largely unknown to the public. Perceptions vary widely, even among experts, leading to a broad range of concerns and opinions regarding the long-term effects, environmental impact, and toxicity of these nanotechnology-based products (Bosetti & Vereeck, 2011). Lack of communication and insufficient information can lead to mistrust, suspicion, and even fear. This has the potential to threaten the future of nanotechnology as a whole and could lead to the rejection of specific nanomedical projects. There are some positive aspects to the situation, though. Through a partnership between the public, government, and business, a corporation in Texas, USA called “Nova Centrix” has raised $25 million over the last nine years with the goal of promoting the development of goods based on nanotechnology for the benefit of society as a whole (Kaur *et al.*, 2014).

## FUTURE ASPECTS

Although polycystic ovary syndrome (PCOS) is linked to several chronic health problems, little is known about how it affects women’s health over time. Numerous issues, including as exhaustion, stress, mood swings, diabetes, heart disease, weight gain, and an elevated chance of miscarriage, have been linked to PCOS in women, according to studies. Choosing the most effective treatments for women in the postmenopausal stage remains challenging. Furthermore, research has shown that women with PCOS tend to have higher bone mineral density. Long-term studies are required to better understand the health risks associated with aging in women with PCOS. It’s also critical to identify associated risk factors and genetic alterations (Barthelmess & Naz, 2014). The review includes several herbs that are considered safe and effective natural treatments for polycystic ovarian syndrome (PCOS). Since long-term usage of synthetic drugs is frequently linked to several adverse side effects, it is advised to gradually reduce these risks by using natural therapies to treat PCOS symptoms. Using natural products over the long term is suggested as a safer alternative (Ghani *et al.*, 2019). Exploring natural remedies for PCOS holds significant potential for personalized and holistic treatment. As modern science integrates with traditional practices, herbal medicines are becoming increasingly viable for addressing the complex nature of PCOS. Research is focusing on herbs that can help regulate hormones, improve insulin sensitivity, and offer anti-inflammatory effects, all of which may provide therapeutic benefits in managing PCOS symptoms. Tailored herbal treatments offer the advantage of personalized care, recognizing the diverse ways in which PCOS can manifest in different individuals. However, to determine the efficacy, safety, and appropriate usage of herbal medicines, thorough scientific research is necessary. Integrating herbal treatments with traditional medicine and incorporating them into comprehensive treatment plans, along with patient education, could lead to a more effective and advanced approach to managing PCOS in the future. The aryl hydrocarbon receptor (AhR) has been implicated in the pathogenesis of PCOS in recent investigations (Kunitomi *et al.*, 2021; Serra *et al.*, 2023; Shi *et al.*, 2024). AhR is a key regulator of changes in the function and activity of the hypothalamic paraventricular nucleus and plays a critical role in the interactions between ovarian granulosa cells and oocytes (Anderson *et al.*, 2025). Like many other disorders, PCOS is linked to changes in the gut flora and increased gut permeability. Eighty percent of women with PCOS who are more obese have the most severe gut dysbiosis and permeability problems (Martínez-García *et al.*, 2024). Digestive dysbiosis is regularly linked to a reduction in the short-chain fatty acid butyrate (Cui *et al.*, 2023) which functions as a histone deacetylase inhibitor and improves mitochondrial function (Anderson & Maes, 2020). Numerous herbal substances have been investigated for their effects on permeability and gut flora, frequently raising butyrate levels. Among these are curcumin (Sun *et al.*, 2024), saffron (Peng *et al.*, 2023), nettle (Fan *et al.*, 2021) and ginger (Shen *et al.*, 2024). Butyrate offers numerous benefits, including the upregulation of the melatonergic pathway (Jin *et al.*, 2016), with melatonin being beneficial in managing PCOS (Patel *et al.*, 2023). It is found in various plants and is regulated by a range of phytochemicals. Herbal nanotechnology holds significant potential for treating PCOS by combining advanced nanoscience with traditional herbal medicine. Nanotechnology can enhance the effectiveness and targeted delivery of herbal ingredients, allowing for more personalized treatments of PCOS symptoms. These innovations can be applied in various forms, including devices, bioadhesives, microparticles, nanoparticles, and micropatches. By incorporating nanoparticles into herbal formulations, bioavailability can be improved, leading to better absorption and utilization of the therapeutic compounds. To further improve the therapeutic effects, herbal extracts can also be delivered directly to the ovaries or other afflicted tissues using nanocarriers. This approach could enable more tailored treatments that align with the specific characteristics of PCOS, using herbal nanomedicines. Nevertheless, additional investigation is required to assess the long-term impacts, safety, and bioavailability of herbal nanotechnologies. Collaboration among herbalists, nanotechnologists, and medical professionals will be crucial to advancing this cutting-edge approach to PCOS treatment. Medicinal plants such as *Trigonella foenum-graecum, Cimicifuga racemosa, Vitex agnus-castus*, and cinnamon species have shown potential in managing PCOS. However, because of drawbacks including small sample sizes and brief study periods, their efficacy is still unknown. More preclinical and clinical research with bigger sample sizes and more exacting procedures is required to gain a better understanding of the safety and pharmacological actions of these herbs in PCOS. Additionally, comprehensive research into the reproductive endocrinological effects of these herbal extracts is necessary to fully understand their mechanisms of action and any potential side effects in the treatment of PCOS.

## CONCLUSION

From youth until premenopause, women with PCOS face several challenges throughout their lifetimes, such as infertility, metabolic disorders, and cardiovascular disease. Although synthetic drugs are effective in treating the severe side effects of PCOS raise concerns regarding their long-term viability. Herbal therapy is therefore gaining traction as a substitute to boost acceptance and recovery rates. Clinically validated botanicals can help control PCOS, according to research. However, it is incorrect to assume that herbal medicines are inherently safe and free of side effects. Herbs can cause a range of negative reactions, some of which are serious or even fatal. Both the use and popularity of herbal remedies worldwide are expanding at an exponential rate, as are reports of negative side effects. The idea that “natural” means “safe” is refuted by this fact. As a result, international regulatory policies pertaining to herbal medicines must be standardized and strengthened. Regulating bodies must act proactively to ensure that all herbal medications approved for sale meet strict safety and quality standards to safeguard the public’s health. In conclusion, herbal remedies include hazards even though they provide intriguing potential for PCOS treatment. The notion that natural products are inherently safe must be refuted, and the possibility of disastrous side effects must be acknowledged.
